# Supplementary material for: SyNDI: synchronous network data integration framework
Source: BMC Bioinformatics. 2018 Nov 6;19:403. doi: 10.1186/s12859-018-2426-5 (PMC6219086; doi:10.1186/s12859-018-2426-5)
Supplement: Supplementary file 5 — Files of the example presented in Additional file 4. This additional file is a zip package that contains all files used in Additional file 4. Detailed descriptions of each of these files are presented in a README file included in this zip package. (GZ 30411 kb) [file 12859_2018_2426_MOESM5_ESM.gz › SyNDITools/Meme2Fimo/meme_4.9.0_4.tar.gz!/meme_4.9.0/doc/mhmms.xml]

mhmms


# mhmms -- not currently supported

**Usage:** mhmms [options] <*HMM file*>
<*FASTA file*>

**Description:**

`mhmms` searches a sequence database using a
Meta-MEME motif-based hidden Markov model (HMM) of the kind
produced by mhmm. Each sequence in the
database is assigned an E-value, and the IDs and scores of
sequences scoring below a given threshold are printed in sorted
order.

The E-value of a given sequence is the expected number of
sequences which match the given model as well or better than this
sequence that you would expect to see by chance in a random
database of the same size as the given database. Scores are
assigned using a local search algorithm; in other words, the
algorithm finds the subsequence that matches a subset of model
states with highest log-odds.

The emission probabilities in the model are converted to
log-odds scores before performing the local search. This is done by
combining pseudocount probabilities derived from a score matrix
(see the '--pam' and '--score-file' options below) with the
emission frequencies. You can control the relative weight placed on
the emission probabilities versus the pseudocount probabilities
(see '--pseudo-weight' below). The adjusted emission probabilities
are then converted to odds by dividing by background probabilities
(see '--bg-file' below). Finally, they are converted to log-odds
scores by taking their logarithm.

Transition probabilities are converted to log-odds scores by
taking their logarithms before searching. This can be overridden
and the gap scores can be set explicitly using the '--gap-open' and
'--gap-extend' switches, below. This allows you to specify a single
affine gap cost function for all spacers in the model.

**Input:**

- <*HMM file*> - An HMM in Meta-MEME
  format must be provided. If the filename is given as '-', then
  `mhmms` will attempt to read the HMM from standard
  input.
- <*FASTA file*> - `mhmms` requires a
  sequence database in FASTA format.
  If the filename is given as '-', then `mhmms` will
  attempt to read the database from standard input.

**Output:**

The `mhmms` output has up to three sections containing
your search results:

- Database Search Results
- Alignments
- Motif Diagrams

The second two sections will not be present unless the -fancy
option was specified.

The results in all three sections are sorted by increasing
E-value if possible, or by decreasing alignment score if E-values
could not be computed.

**DATABASE SEARCH RESULTS**

The "Database Search Results" section consists of lines of the
following form:

`<ID> <E-value> <Score>
<Start> <End> <Length>
<Description>`

These fields contain, for each alignment,

- **ID** - The sequence identifier, as given in the database
  file.
- **E-value** - The E-value, which is the total number of
  alignments that you would expect with alignment scores as good as
  this alignment if the database contained only sequences unrelated
  to the query. Thus, a small E-value indicates a good
  alignment.
- **Score** - The Viterbi alignment score. The Viterbi
  algorithm finds the (local) path through the model that has the
  highest score with respect to the given database sequence. This
  probability is divided by the probability of the same sequence with
  respect to a simple, zero-order Markov background model. The
  resulting ratio (called the odds score) is converted to a logarithm
  (base 2) and is printed in this column. The Viterbi log-odds score
  is the basis for the E-value score in the previous column.
- **Start** - The position in the sequence where the alignment
  begins.
- **End** - The position in the sequence where the alignment
  ends.
- **Length** - The length of the sequence.
- **Description** - The sequence descriptor. This description
  is taken from the FASTA database file, and is truncated so that the
  output fits easily on one line.

**ALIGNMENTS**

Each alignment lists the sequence identifier, alignment E-value
and log-odds score along the left. On the right, it shows the
alignment of the model with the sequence in groups of three
segments.

- Each segment contains, in the bottom-most line, 50 letters from
  the target database sequence, flanked by that segment's start and
  end locations within the entire sequence. Thus, the first segment
  would be flanked by \"1\" and \"49,\" the second by \"50\" and
  \"99,\" etc.
- Aligned above these 50-letter segments are the motifs in the
  model. The motifs are labeled with numbers in the order they
  appeared in the `MEME` output file from which the model
  was built. A plus or minus sign preceding a motif indicates that
  the motif occurs on the given (+) or reverse complement (-) of the
  DNA sequence in the database. Each position within a motif region
  is indicated by a letter, and each gap position is indicated with a
  period.
- In between the sequence segment and the corresponding model
  positions is a line that indicates the degree of match between the
  motifs and the sequence. If the letter in the motif with the
  largest log-odds score appears in the sequence, then the match row
  contains that letter. If the sequence letter does not have the
  largest log-odds score, but does have a positive log-odds score,
  then the match row contains a plus sign. Otherwise, the match row
  is empty.

**MOTIF DIAGRAMS**

The motif diagrams section shows the alignments in schematic
format. For each alignment, in the right two columns, it shows the
sequence identifier and the alignment E-value. On the left, it
shows the positions and spacings of the motifs in the alignment.
Hits are labeled with numbers corresponding to the order the motifs
were given in the query. A plus or minus sign preceding a motif
indicates that the motif occurs on the given (+) or reverse
complement (-) of the DNA sequence in the database.

**LOG-ODDS SCORES**

The log-odds scores for each motif column are created using
prior information on the letters appearing in alignment columns.
The prior information is the target frequencies [Karlin,S. and
Altschul,S.F., PNAS USA , 87, 2264-2268] implicit in a scoring
matrix. Meta-MEME can read a user-specified scoring matrix (in the
same format as used by the BLAST family of programs) from a file or
generate a PAM matrix. By default, PAM 250 is used for proteins,
and PAM 1 is used for DNA. For DNA, the "PAM 1" frequency matrix
is

```
              .990 .002 .006 .002
              .002 .990 .002 .006 
              .060 .002 .990 .020
              .020 .060 .002 .990
```

Meta-MEME calculates the target frequencies q_ij_ =
p_i_p_j_ exp(L s_ij_) from the scoring
matrix s_ij_ and the background letter frequencies
p_i_ by finding the value of L that makes the
q_ij_ sum to one. These target frequencies are then used
to create pseudo-frequencies to be added to the emission
frequencies of the column, following the approach of [Henikoff,S.
and Henikoff,J.G., JMB, 243, 574-578]. The pseudo-frequency for the
i^th^ letter is computed as: g_i_ = sum _j in
alphabet_ (f_j_ q_ij_/p_j_).

The pseudo-frequencies, g_i_, are then combined with
the emission frequencies, f_i_ to give frequency
estimates

Q_i_ = (alpha f_i_ + beta g_i_)
/ (alpha + beta).

In general, alpha should be proportional to the amount of
independent information in the emission frequencies. We have set it
to the constant 20. The parameter beta is arbitrary and controls
the relative importance of prior information. We set it to the
constant 10.

Our method is essentially that used in PSI-BLAST [Altschul,S.F
et al., NAR, 25:17, 3389-3402] without

1. sequence weighting, and
2. scaling for amount of independent information (alpha).

To do 1) and 2) correctly would require having and using alignment
information rather than emission frequencies as the starting
point.

**Options:**

- --paths single|all - This option determines how
  `mhmms` computes raw scores. With the
  `single` option, `mhmms` computes the Viterbi
  score, which is the log-odds score associated with the single most
  likely match between the sequence and the model. The
  `all` option yields the total log-odds score, which is
  the sum of the log-odds of all sequence-to-model matches. The
  default is Viterbi scoring.
- --p-thresh <p-value threshold> - The '--p-thresh' switch
  activates p-value score mode with the given threshold. (The default
  score mode is called "log-odds score mode".) In p-value score mode,
  motif match scores are converted to their p-values. They are then
  converted to bit scores as follows:

  S = -log_2_(p/T)

  where S is the bit score of the hit, p is the p-value of the
  log-odds score, and T is the p-value threshold. In this way, only
  hits more significant than the p-value threshold get positive
  scores. The p-value threshold, T, must be in the range
  0<T<=1. This mode of scoring automatically activates the
  '-motif-scoring' feature (described below under "Advanced
  Options:") so that partial motif hits are disallowed.

  **Note**:

  - If *p-value threshold* is too small, there may be few
    (or no) "hits", and, consequently, few (or no) matches. This may
    cause `mhmms` to be unable to compute match E-values, or
    to report no matches. Small values of the *p-value
    threshold* may also cause the reported E-values to be
    inaccurate. In this case, the E-values will always be too large
    (conservative). The proper value for the *p-value threshold*
    can only be determined by experimentation since it depends on the
    number of motifs, the information content of the motifs and the
    value of *maxgap*.
- --both-strands - This allows matches to occur on either DNA
  strand. By default, motif matches are only on the given strand. The
  -both-strand switch implies the motif-scoring switch.
- --e-thresh <*threshold*> - `mhmms` lists
  the sequences that have E-values below the given threshold. The
  default threshold is 10.
- --fancy - The '--fancy' switch turns on a more detailed output
  format that shows, in addition to the score for each sequence, the
  complete model-to-sequence match. Here is an example of the fancy
  output format, showing a two-motif model matching a sequence of
  length 179:
- ```
                           *.................................................
                                                                             
          170K_TRVPS     1  GAHLVPTKSGDADTYNANSDRTLCALLSELPLEKAVMVTYGGDDSLIAF    49

                           .........................FDWqKFAGtWH..............
                                                     D+  F G+                
          170K_TRVPS    50 PRGTQFVDPCPKLATKWNFECKIFKYDVPMFCGKFLLKTSSCYEFVPDPV    99

                           ..................................................
                                                                             
          170K_TRVPS   100 KVLTKLGKKSIKDVQHLAEIYISLNDSNRALGNYMVVSKLSESVSDRYLY   149

                           ......GYCPEVKPI...............*
                                   C+  K I                
          170K_TRVPS   150 KGDSVHALCALWKHIKSFTALCTLLPRRKG    179
  ```
- --width <*int*> - Specify the width (in characters)
  of each line in the output. The description of each sequence, which
  is taken from the input FASTA file, will be truncated as necessary.
  By default, the output width is 132 characters.
- --nosort - Do not sort the output.
- --bg-file <*file*> - This switch allows you to
  specify a different background letter distribution. The background
  letter distribution is used in converting the emission
  probabilities in the model to log-odds. By default, the background
  letter distribution of the appropriate (DNA or protein) NCBI
  non-redundant database is used. With the '--bg-file' switch, the
  background distribution is read from a file whose format is the
  same as used by MEME and MAST. The file must contain one line for
  each letter in the (unambiguous) character set. Each line must
  contain the letter followed by the letter's frequency. All other
  lines in the file are ignored, including comment lines starting
  with '#'. For example, *file* might contain:

  ```
  # tuple   frequency_non_coding
        a       0.324
        c       0.176
        g       0.176
        t       0.324
  ```

  (You can produce a file in the proper format from any FASTA
  sequence file (DNA or protein) using the fasta-get-markov utility,
  which is included with the Meta-MEME distribution in directory /bin.
  Type fasta-get-markov < f.fasta > f.bg to make a file named
  "f.bg" containing the letter distribution from FASTA file
  "f.fasta". )
- --allow-weak-motifs - In p-value score mode, weak motifs are
  defined as ones where the best possible hit has a p-value less than
  the p-value threshold. Such motifs cannot contribute to a match in
  p-value score mode. By default, `mhmmscan` rejects any
  search containing weak motifs, unless the -allow-weak-motifs switch
  is given. In that case, the search will proceed, but the weak
  motifs will never appear in any matches. **Note**:This switch
  only applies to p-value score mode.
- --progress <value> - Print to standard error a progress
  message after every *value* sequences.
- --verbosity 1|2|3|4|5 - Set the verbosity level of the output
  to stderr. The default level is 2.
- --noheader - Do not put a header on the output file.
- --noparams - Do not list the parameters at the end of the
  output.
- --notime - Do not print the running time and host name at the
  end of the output.
- --quiet - Combine the previous three flags and set verbosity to
  1.

**Advanced Options:**

- --zselo - Specifying the '--zselo' switch causes the spacer
  emission log-odds scores to be set to zero. This prevents regions
  of unusual base/residue composition matching spacers well when the
  spacer emission frequencies are different than the background
  frequencies. It is particularly useful with DNA models.
- --gap-open <*cost*> - The '--gap-open' switch causes
  **all** transitions into a spacer state to be assigned a
  log-odds score equal to *-cost*. Together with the
  '--gap-extend' switch, this allows you to specify an affine gap
  penalty function, overriding the gap penalty implicit in the model
  (transition probabilities into and out of gap states).
- --gap-extend <*cost*> - The '--gap-extend' switch
  causes **all** spacer self-loop log-odds scores to be set to
  *-cost*. In addition, it causes all other transitions out of
  a spacer to be set to zero. Together with the '--gap-open' switch,
  this allows you to specify an affine gap penalty function,
  overriding the gap penalty implicit in the model (self-loop
  transition probabilities of gap states).

*The following option is automatically invoked when you
specify --p-thresh. You can also set it when you do not want
p-value score but want to prevent partial matches to
motifs.*

- --motif-scoring - This option prevents alignments from
  containing a partial motif.

*The following options can be used in both p-value and
log-odds score modes to control how the emission probabilities in
the HMM are converted into log-odds scores.*

- --pseudo-weight <*weight on pseudocounts*> - By
  default, the pseudocount probabilities are weighted by 10, and
  emission probabilities in the model by 20. The weight on the
  pseudocount probabilities can be adjusted to any value greater than
  or equal to zero using the '--pseudo-weight' switch. The smaller
  the weight, the less effect the pseudocount probabilities have, and
  the closer the adjusted probabilities will be to the emission
  probabilities in the model.
- --pam <distance> - By default, target probabilities are
  derived from the *distance-250* PAM matrix for proteins, and
  from a *distance-1* transition/transversion matrix for DNA.
  With the '-pam' switch, you can specify a different **integer**
  distance from 1 to 500. (This can be overridden with the
  '--score-file' switch, below). The *distance-1*
  transition/transversion joint probability matrix for DNA is given
  below:

  ```
               A    C    G    T    
          A  .990 .002 .006 .002
          C  .002 .990 .002 .006
          G  .006 .002 .990 .002
          T  .002 .006 .002 .990
  ```
- --score-file <*score file*> - The '--score-file'
  switch causes a score file (in BLAST format) to be read and used
  instead of the built-in PAM (for proteins) or
  transition/transversion (for DNA) score file. Several score files
  are provided (including BLOSUM62) in directory mhmm/data. Other,
  user-provided score files may be specified as well, as long as they
  are in the proper format.

**Bugs:** None known.

Author: William
Stafford Noble.
